# Supplementary material for: Penalized ensemble Kalman filters for high dimensional non-linear systems
Source: PLoS One. 2021 Mar 18;16(3):e0248046. doi: 10.1371/journal.pone.0248046 (PMC7971544; doi:10.1371/journal.pone.0248046)
Supplement: Attachment — Submitted filename: REV-PONE-D-20-14752.pdf [file pone.0248046.s001.pdf]

REVIEWER COMMENTS FOR PONE-D-20-14752

After reading the paper Penalized Ensemble Kalman Filters for High Dimensional Non-linear Systems, according to authors, the paper proposes to exploit the conditional independence of model components concerning model dynamics (which should be implicitly captured into the LASSO coefficients). I have some major concerns with regard to this paper,

1. I really did not like your paper's organization, how an "Other Work" section can be before the Section wherein you propose your method? The paper should follow a standard outline: Introduction, Preliminaries (here you can add the "Other Work" section, I suggest to rename it as "Related Work" or something like that), Proposed Method (Derivation, Computational Cost, Potential Parallel Implementation, etc.), Experimental Settings, and Results.

2. Your abstract makes a claim which (from a very theoretical point of view) is impossible: "it can be theoretically proven that the PEnKF will have good performance (the error will converge to zero) despite having fewer ensemble members than state dimensions." The errors of what? Both sources of information in EnKF formulations are uncertain, how is it possible (I guess you meant posterior errors) that the errors will converge to zero? On the other hand, errors can accumulate among assimilation steps! So, there are many manners in which errors can develop across assimilation cycles.

3. From a fundamental point of view, the authors propose an estimate of the precision covariance but, the employ the dual formulation of the EnkF (wherein analysis incremnets are computed onto the observation space). This formulation relies on an information matrix of the form (R+H*P*H') and not on P (the background error covariance matrix) inverse. It would be best if you used the (inv(P) + H'*inv(R)*H) formulation.

4. Following 2, this means that, the filter update does not depend on the precision matrix of background errors. How do you map your estimate P inverse to P?

5. In the abstract, one reads: "These theoretical results are supported with simulations of several non-linear and high dimensional systems" but, you work with only two models, the Lorenz-96 (40 state variables) and a modified SWE (900 state variables). They are not really that "high dimensional".

6. The computational cost of your method is very expensive; it involves the square of the number of state variables, making it unpractical under current operational Data-Assimilation scenarios. Besides, to briefly talk about parallel implementation relying on some parallel solver, it isn't a good idea. You must show how parallel linear algebra solvers can be implemented for your method (i.e., how you split the model domain and share information across processors belonging to some local neighborhood...).

7. The English must be improved across all paper.

On the other hand, one of my strong concerns: I do not see what's the advantage (and the difference) of your method and that proposed by Nino-Ruiz (you cited a conference paper), taking a close look of this method:

1. Nino-Ruiz, E. D., Sandu, A., & Deng, X. (2018). An ensemble Kalman filter implementation based on modified Cholesky decomposition for inverse covariance matrix estimation. SIAM Journal on Scientific Computing, 40(2), A867-A886.
2. Nino-Ruiz, E. D., Sandu, A., & Deng, X. (2019). A parallel implementation of the ensemble Kalman filter based on modified Cholesky decomposition. Journal of Computational Science, 36, 100654.

it seems to be the same that your proposal, right? Based on all my previous comments, **I suggest rejecting the paper at this time.**
