# Supplementary material for: Penalized ensemble Kalman filters for high dimensional non-linear systems
Source: PLoS One. 2021 Mar 18;16(3):e0248046. doi: 10.1371/journal.pone.0248046 (PMC7971544; doi:10.1371/journal.pone.0248046)
Supplement: Attachment — Submitted filename: Response to Reviewers.pdf [file pone.0248046.s002.pdf]

We would like to thank the reviewers for the helpful comments. Our responses are in blue below.

Reviewer #1: This is a very interesting paper on reducing noisy covariance estimates in Ensemble Kalman filters. The paper is well-written in general, but it is perhaps too easy to lose non-mathematicians. Assuming this new scheme is meant to be used in practice the authors should provide an easier access for practitioners. Furthermore, the claims on its applicability for real-world systems are perhaps overstated. I suggest a modification of the paper along the following lines.

1. Explain in the abstract how the new filter is different from localization. At the moment the abstract contains nothing new.

We have modified the abstract to summarize the difference.

“Further, as contrasted to localization methods, the proposed approach learns the covariance structure associated with the dynamical system.”

2. On particle filters, I suggest the authors have a look at the latest on particle filtering in high-dimensional systems, such as Van Leeuwen, P.J., L. Nerger, R. Potthast, S. Reich, and H.R. Kunsch (2019) Particle Filters for Geoscience applications: A Review, Q.J.Royal Meteorol. Soc., doi: 10.1002/qj.3551. No need to reference it, but there is more than equal weights and mixtures with EnKF in the particle filter literature.

The cited paper by the reviewer is a very interesting recent review of particle filters of which we were not aware. We have included it to have a more complete reference to particle filters. Thank you for bringing it up.

3. Lines 70-95: The fact that a conditionally sparse correlation matrix has a sparse inverse is extremely important and a fact I wasn't aware of, as I'm sure many in the data-assimilation community are not. Please provide proof or reference.

This fact is indeed very important and, although it is well known in multivariate statistics, we agree that it may not be as well known to the PLoS One audience. We have added a citation [42] to it in the revision.

[42] Lauritzen SL. Graphical Models. In: Graphical Models. Oxford; 1996. p. 129

4. Algorithm 1 still uses perturbed observations, while it has been shown that perturbing H(x) is the correct thing to do, and the better thing to do when the system is nonlinear, see e.g. Van Leeuwen, P.J., (2020) A consistent interpretation of the stochastic version of the Ensemble Kalman Filter, Q.J. Royal Meteorol. Soc., doi: 10.1002/qj.3819

Indeed, the proposed Algorithm 1 uses perturbed observations, which, under assumptions of linear H and Gaussian noise, is equivalent to perturbing H(x). The paper

Leeuwen 2020 establishes that perturbing the model H(x) is better than perturbing the observations when observation errors are not Gaussian and the observation operator H is nonlinear. Specifically, Van Leeuwen (2020) shows that in the regime where the prior is symmetric and the likelihood is skewed, the perturbed observations approach leads to posterior samples with skewness having the wrong sign. However, our paper is concerned with the scenario where the dynamical system equations are non-linear, but the measurement equations are (near) linear with Gaussian measurement errors. Hence, Van Leeuwen's conclusions do not directly apply to our regime. We have added additional clarification of this in the revision in addition to a citation of this article. We point out to the reviewer that Van Leeuwen's paper appeared after we submitted our paper to PLoS One.

5. Alg 1: Perhaps mention here that an EnKF scheme will not need to calculate P^f explicitly in high-dimensional systems but instead works with the ensemble matrix of size n*p.

This is a very good comment. We have added a discussion of computational complexity of the regular EnKF to the computation section 3.3, which is the appropriate place to discuss this.

6. Please number all equations. In the equation below eq 1, please define B^{\lambda}, it seems that it is a Bergman divergence and the equation below eq (1) is actually its definition? Furthermore, the appendix is missing, or is the 'Definition' the start of the appendix? . I suggest to expand section 2.2, it is most important!

We have added numbers for all the equations. We have defined the Bregman divergence as B in eq 1 and the empirical Bregman divergence with penalty (eq 2) as \hat{B}^\lambda (where lambda refers to the penalty parameter). Could the reviewer clarify or suggest which parts of section 2.2 should be expanded upon to provide a more complete overview?

We have fixed the issues with the appendix section header not showing up.

7. Define the deformed = sign in Theorem 1. Note PLOS ONE is not a maths or stats journal, but tries to be broader than that. What is a sub-Gaussian ensemble?

We appreciate the reviewer's comment and understand that PLOS ONE is not a math journal and have therefore supplied additional explanations of mathematical concepts in the revision. See below.

In the revision we have added a definition of the symbol to the explanation just below Theorem 1.

A sub-Gaussian ensemble is an ensemble of distributions that are sub-Gaussian. A sub-Gaussian distribution is any distribution whose tails decay faster than a Gaussian distribution, which is equivalent to a bounded moment condition, specifically the p-th

root of the p-th order moment decays at least as fast as K\sqrt(p) for a constant K<\infty [47]. The sub-Gaussian assumption is a standard assumption in probability that is used to obtain convergence and convergence rates like we do in Theorem 1. Many practical distributions satisfy this assumption, e.g. the Gaussian distribution, the Rademacher distribution, and the distribution of any bounded random variable. In the revision we have added the following additional clarifying remarks:

“Additionally, the rate at which the estimator converges depends on the distribution of the ensemble members. For example, if the ensemble members follow a light tailed distribution, more specifically a sub-Gaussian distribution [47], the estimator will typically have a faster rate of convergence than if the ensemble follows a heavy tailed distribution.”

[47] Vershynin R. High-dimensional probability: An introduction with applications in data science. Cambridge University Press; 2018.

8. line 175: p is the dimension of the state, not the number of states.

Thank you for noticing the typo. We have fixed this.

9. lines 183-187: The authors seem to forget that the number n needed depends strongly on the system dynamics: in simple terms n should be of the size of the growing and neutral modes in the dynamical system, simply because the decaying modes do not hamper a forecast. See e.g. SIAM/ASA J. Uncertainty Quantification, 5(1), 304–333. Degenerate Kalman Filter Error Covariances and Their Convergence onto the Unstable Subspace Marc Bocquet, Karthik S. Gurumoorthy, Amit Apte, Alberto Carrassi, Colin Grudzien, and Christopher K. R. T. Jones https://doi.org/10.1137/16M1068712

The reviewer raises an important point about the need to select n dependent on the modes of the system dynamics. These modes are embedded in eigenvalues of the true forecast covariance matrix, P^f in our notation. The modes do not explicitly appear in our Theorem 1 since it is stated as an asymptotic, large n, p limit in order to obtain conditions on n,p and sparsity parameter d that guarantee convergence. We think that with additional work, a refined asymptotic analysis would be able to reveal convergence rates and it is here that the role of the eigenvalues of P^f of would appear, allowing a comparison to Bocquet (2020). However, the effort required to do this would be substantially beyond the scope of the paper. This is due to the following complications: 1) the analysis in Bocquet (2020) is for estimation of the error covariance matrix while our analysis is for estimation of the inverse of this matrix. 2) unlike Bocquet (2020), our analysis introduces a sparsity assumption. 3) unlike Boquet (2020), our analysis holds for non-linear system equations. In the revision we have included a reference to Bocquet (2020) in addition to a discussion of the possibility to refine our analysis.

10. Line 188: Pr(..) is typically used to denote probability, while the authors mean probability density here. Perhaps change notation.

You are correct. We have changed the notation from Pr(…) to p(…).

11. Section 3.3: what is q? Is that r?

Yes, q is supposed to be r, which is the dimension of the observations y. We fixed the typos.

12. Section 3.3: On the storage, assume the computer can store 100*dim(x) variables (that is reality for numerical weather prediction). And suppose we have 10,000 blocks. Than a matrix of size (p/k)*(p/k) = 10,000 dim(x)^2 /10^8 = dim(x)^2/10^4. For a typical system dim(x) = 10^9 or 10^10, so there is no way one can store this. In fact, even s can be too large to store. That is why all ensemble methods work with the ensemble matrix of size n*p. Please add something like this to the discussion of the computational burden.
We have added additional discussion of the storage burden compared to the EnKF in section 3.3 following the discussion of computational complexity, which was suggested in comment 5.

13. Perhaps provide pseudo code for the new filter. That would help sell it strongly...!

Thanks for the suggestion. We have moved the original EnKF algorithm to the appendix as it is just background information and added pseduo code for our filter in section 3.

14. Please describe what is shown in fig 1 more accurately. The caption is confusing and incomplete, and the quality of the figure is not ok. Furthermore, spurious correlations are still 5%, which would ruin a weather forecast. Please comment.

We have clarified the caption and fixed the resolution of the figures. With the higher quality figures the reviewer will see that the small fluctuations in the tails of the lines are not 5%, but much smaller (around 1% on average).

15. Fig 2: How large is n? (This is a remarkable result, great!)

n is 25. We have added this in the caption. Thanks for the positive comment.

16. Section 4.2: Please provide model equations, e.g. in an appendix.

We have added the model equations in the appendix as S1-S3.

17. Section 5: Can the authors explain the use of the word 'learning' when discussing the PEnKF? I seem to be missing how information from one data assimilation step is

propagating to the other, other than via the ensemble itself. All methods have that in common, so that cannot be the learning part.

We have added additional clarification. Essentially by “learning” we mean that we use a data-driven non-parametric estimator \tilde{K} instead of a model-based estimator K as in the standard EnKF. Learning occurs since we don’t make any assumption about the conditional correlations between states, but instead let the data define them.

18. Line 375: There are many ways to infer the performance of a data assimilation system applied to real systems. And there is always a lot of scientific knowledge about the system under study. Because of this, I find the 'no ground truth' argument less convincing. More convincing is that despite this knowledge a lot of tuning is needed to make things work. The PEnKF might reduce the amount of tuning needed. And please don't use the wording 'oracle information'; as mentioned we always have prior information so we should always use it.

We have revised this argument to restrict the scenario to the case where there is less prior knowledge or there is noisy prior knowledge. While there is a lot of scientific knowledge about real systems, there are often simplifying assumptions that may not hold exactly. Furthermore, it can be risky to impose hard constraints to a system when the knowledge about the system is noisy as this will force the model to incorporate incorrect information.

“Through simulations, we show that the PEnKF can do at least as well as, and sometimes better than, localized filters that use much more prior information. We emphasize that by doing just as well as the B-Loc EnKF which has a close to optimal taper matrix, the PEnKF is effectively correctly learning the structure of interactions

between the state variables. Thus the PEnKF is able to infer from the ensembles the true conditional correlation between the states as opposed to having it or the correlation pre-defined. In a non-simulation setting where there is possibly more uncertainty in the prior knowledge of the interactions between state variables, correct localization is much more difficult, making any localized filter’s performance likely sub-optimal.”

19. Following on from the previous point, the PEnKF also needs tuning of the lambda matrix for the L1 optimization. How is that different from tuning a localization lengthscale?

The reviewer is correct that PEnKF requires tuning of the sparsity parameter lambda. Thus there is little difference between tuning lambda vs tuning a localization lengthscale – one could apply the proposed bootstrap tuning procedure to both cases. The benefit of the PEnKF is not that there is no need for tuning parameters, but that there are fewer structural assumptions (localization in additional to the lengthscale parameter needs the structure of how the covariance matrix decays). Thus, following the argument in the

previous comment, the parameter we tune does not impose structural knowledge that could lead to sub-optimal filter performance if incorrect.

20. Line 385: One has to be careful, the authors mention that the PEnKF has a slight implicit inflation but in their comparison they do not allow other methods, that do not have this implicit inflation, to have some explicit inflation. It is practical knowledge that a slight inflation can often help any EnKF tremendously. So one might consider the comparison somewhat unfair. Also, note that most operational weather prediction run with some kind of adaptive inflation method based on the performance of the data assimilation.

It is true that inflation can help a EnKF based model. We did not add inflation to the labeled B-Loc EnKF (previously labeled TAPER-EnKF) in order to have comparable statistics with the results reported in as many other papers as possible. However [34] compares their model NLEAF to a model that has added inflation (and shows better performance of their NLEAF model), and we show comparable results in Table 1 to the NLEAF model. Thus we believe we would still have comparable if not better performance even against models with some added inflation.

21. Proof of Theorem 1: Replace 'term' by 'factor'.

We have fixed this.

Reviewer #2:

1. I really did not like your paper's organization, how an "Other Work" section can be before the Section wherein you propose your method? The paper should follow a standard outline: Introduction, Preliminaries (here you can add the "Other Work" section, I suggest to rename it as "Related Work" or something like that), Proposed Method (Derivation, Computational Cost, Potential Parallel Implementation, etc.), Experimental Settings, and Results.

We have changed “Other Work” to “Related Work”. Otherwise we are indeed following the standard outline suggested with more descriptive titles that reflect the content in each section.

2. Your abstract makes a claim which (from a very theoretical point of view) is impossible: "it can be theoretically proven that the PEnKF will have good performance (the error will converge to zero) despite having fewer ensemble members than state dimensions." The errors of what? Both sources of information in EnKF formulations are uncertain, how is it possible (I guess you meant posterior errors) that the errors will converge to zero? On the other hand, errors can accumulate among assimilation steps! So, there are many manners in which errors can develop across assimilation cycles.

We have clarified that we mean the estimation error in the abstract.

“Under certain conditions, it can be theoretically proven that the PEnKF will be accurate (the estimation error will converge to zero) despite having fewer ensemble members than state dimensions.”

3. From a fundamental point of view, the authors propose an estimate of the precision covariance but, the employ the dual formulation of the EnkF (wherein analysis incremnets are computed onto the observation space). This formulation relies on an information matrix of the form (R+H*P*H') and not on P (the background error covariance matrix) inverse. It would be best if you used the (inv(P) + H'*inv(R)*H) formulation.

We have derived the Kalman gain matrix in its dual formulation in Definition 4. To increase clarity, we have also added the equivalent primal form (inv(P)+H’*inv(R)*H)to equation 4.

4. Following 2, this means that, the filter update does not depend on the precision matrix of background errors. How do you map your estimate P inverse to P? 5. In the abstract, one reads: "These theoretical results are supported with simulations of several non-linear and high dimensional systems" but, you work with only two models, the Lorenz-96 (40 state variables) and a modified SWE (900 state variables). They are not really that "high dimensional".

We emphasize that our filter update does directly depend on the precision matrix – it is simply that a sparsity regularized estimator of the precision matrix is used. Our precision matrix estimator is positive definite, so it can be inverted using a standard sparse linear systems solver. By high dimensional, we are using the convention common in high dimensional statistics [1] that the number of state variables p exceeds the number n of ensemble members.

[1] Bühlmann, Peter, and Sara Van De Geer. Statistics for high-dimensional data: methods, theory and applications. Springer Science & Business Media, 2011.

“In addition, because the estimator of the covariance $ \tilde{\mat{P}}^f $ is positive definite, standard matrix inversion techniques can convert it to an estimator of the precision matrix.”

6. The computational cost of your method is very expensive; it involves the square of the number of state variables, making it unpractical under current operational Data-Assimilation scenarios. Besides, to briefly talk about parallel implementation relying on some parallel solver, it isn't a good idea. You must show how parallel linear algebra solvers can be implemented for your method (i.e., how you split the model domain and share information across processors belonging to some local neighborhood...).

Our paper uses a sparsity assumption on the inverse covariance matrix to reduce the computational complexity of estimating this matrix to significantly below the number of state variables squared (s << p). The BIGQUIC algorithm, which is designed for sparse

inverse covariance estimation, is used to numerically solve our objective function, whose solution is a sparse positive definite matrix, in parallel. The actual parallel implementation is thus equivalent to described in the BIGQUIC algorithm paper (reference 49).

7. The English must be improved across all paper.

We are all native English speakers and have corrected the minor typographical errors in the manuscript.

On the other hand, one of my strong concerns: I do not see what's the advantage (and the difference) of your method and that proposed by Nino-Ruiz (you cited a conference paper), taking a close look of this method: 1. Nino-Ruiz, E. D., Sandu, A., & Deng, X. (2018). An ensemble Kalman filter implementation based on modified Cholesky decomposition for inverse covariance matrix estimation. SIAM Journal on Scientific Computing, 40(2), A867-A886. 2. Nino-Ruiz, E. D., Sandu, A., & Deng, X. (2019). A parallel implementation of the ensemble Kalman filter based on modified Cholesky decomposition. Journal of Computational Science, 36, 100654. it seems to be the same that your proposal, right? Based on all my previous comments, I suggest rejecting the paper at this time.

The papers suggested are proposing a low rank estimator whereas we are proposing a sparse estimator. While both are indeed estimators for the inverse covariance matrix, the estimators themselves are completely different. We have updated our references to reflect the later more encompassing journal paper instead of the earlier more preliminary conference one.

Reviewer #3: This manuscript proposes a method which allows the application of ensemble Kalman filters in high-dimensional systems keeping accuracy and stability, and at the same time allowing for realistic ensemble sizes much smaller than the actual size of the system. To do this it uses a penalisation based on ideas from lasso. The idea is find an estimator of the inverse of the covariance matrix subject to some constrains like sparcity. This is

I think this is a very interesting contribution to the KF and DA literature. It is very well written. It presents a good literature review, nice theoretical derivations and some numerical experiments. I think this will be acceptable for publication after some minor reviews.

-- Minor Comments:

- The sub-Gaussian criterion described in line 177 seems quite important for theorem 1. In my first read it was somehow lost, and then I had to go back and read more carefully. Can you emphasise it somehow? Or maybe even state this criterion before the theorem.

We have added additional description before theorem 1 emphasizing that the rate of the estimators convergence is affected by the sub-Gaussian criterion or lack of it.

“Additionally, the rate at which the estimator converges depends on the distribution of the ensemble members. For example, if the ensemble members follow a light tailed distribution, more specifically a sub-Gaussian distribution [47], the estimator will typically have a faster rate of convergence than if the ensemble follows a heavy tailed distribution.”

[47] Vershynin R. High-dimensional probability: An introduction with applications in data science. Cambridge University Press; 2018.

- I would really avoid using the term TAPER-EnKF, since I have never seen it before in the EnKF literature. What you are describing is an EnKF with B-localisation, or localisation in model space.

This is a good point. We have changed this to B-Loc ENKF in the revised paper.

-- Comments:
- In line 234 you mention equation (1) is not a likelihood unless the states are Gaussian. Is it really as restrictive as this? Or can the states belong to certain class of distributions?

We meant that in the special case when the states are actually Gaussian, equation 1, is a penalized likelihood function, but this is not necessary for the Theorem. The Theorem relates the penalty parameter to the rate of convergence of the estimator, but the constant c_lambda still needs to be chosen. Thus, when equation (1) is a likelihood, it can be used to form a valid information criterion (BIC) and we can use it to evaluate our choice in c_lambda. When equation (1) is not a likelihood, it is technically not a valid information criterion, but we believe it can still form a good evaluator for c_lambda.

- Your approach is quite interesting since you focus on the construction of inv(Pf) rather than Pf itself, and you ask from some requirements from inv(Pf).
Let's say you have a real Pf and two estimators: one for Pf and the other for its inverse. How does the sparsity in one relate to the other? I am just reading line 130 where you mention the 'intuition' about inv(Pf) being sparse and diagonal dominant. It is usually this assumption but on Pf what is used. How do both relate?

Sparsity in the covariance does not generally imply sparsity in the inverse, or vice versa, unless the covariance is block diagonal. Sparsity can exist in both but the sparsity pattern is the same only if the covariance is block diagonal. Additional discussion of this point has been included in addition to the citation of the standard textbook on graphical models that covers sparse covariance and inverse covariance models:

Sec. 5.1.3 of Steffen L. Lauritzen, Graphical Models, Oxford, 1996

- In many applications localisation is done differently in different physical dimensions. For instance in NWP models localisation is done horizontally and vertically differently. How would this work under your method?

Because we learn or infer the conditional correlation between the states, this is handled automatically in our method since we learn the associated sparsity pattern. If the actual relationship between states is different in different physical dimensions then this will be learned by our model. For example in our SWE simulation, the relationship between rain and fluid height is not obvious or as easily discernible as sensors on a grid, but we are able to learn the conditional covariances (non-zero elements of sparse inverse covariance) between a rain state and a fluid height one. The description for choosing lambda in Section 4.2 discusses how we separate the parameter matrix by the physical type of the state variables (rain, fluid height, and wind).

- The regularisation parameter of the Bregman divergence function is crucial in the process described here. As I understand, it can be a scalar or matrix. In fact you have used a scalar for L96 and a matrix for SWE and I guess this depends on the 'nature' of the state variables. What would the process to find either an optimal or a useful lambda be in a large model? I am just asking to discuss this a little further, not to do it. Also, can one obtain a physical interpretation of the parameter. With localisation one can speak, for instance, of de-correlation length-scales.

We have added to the discussion at the beginning of the Section 4 where we discuss choosing lambda. It can be either a scalar or a matrix (where the scalar is basically a diagonal matrix of the same value) and the choice does depend on the belief on the "nature" of the state variables e.g. if there are various types of state variables, it is unlikely they would have the same penalty parameter. For larger models, it may be more practical to choose one lambda per type of state variable instead of allowing for a different on for every state, as this leads to fewer free parameters to tune.

We have also divided the section into subsections to further emphasize the importance of choosing lambda.

The penalty parameter can be interpreted as a measure of sparsity of the inverse covariance. A larger lambda implies a sparser \tilde{S}^-1. Furthermore, as\tilde{S}^-1 becomes less sparse as the length scale of conditional dependency increases. It is common in sparse inverse covariance estimation to relate the penalty parameter directly to the number of non-zero elements of the matrix by generating the full "Glasso path" of sparsity vs penalty [1].

[1] Friedman, Jerome, Trevor Hastie, Rob Tibshirani, and Maintainer Rob Tibshirani. "Package 'glasso'." (2015). https://cran.r-project.org/web/packages/glasso/
