# Supplementary material for: Penalized ensemble Kalman filters for high dimensional non-linear systems
Source: PLoS One. 2021 Mar 18;16(3):e0248046. doi: 10.1371/journal.pone.0248046 (PMC7971544; doi:10.1371/journal.pone.0248046)
Supplement: Attachment — Submitted filename: Response to Reviewers.pdf [file pone.0248046.s003.pdf]

We would like to thank the Editor for handling our paper and the reviewers for their helpful comments. We have responded to all of these comments and suggestions and our responses to the reviewers are shown in blue below.

Reviewer #1: The authors did a very good job in replying to the issues raised, and I suggest acceptance after the following minor comments have been taken into consideration:

We are gratified by the reviewer's positive comments acknowledging the responsiveness of our revisions of the paper.

Line 200: number of ensemble members

Line 203: the order of ... Please fix notation.

Line 408: There is only one ensemble.

Second reference: The second author is P.J. Van Leeuwen

We have fixed the above typos. Thank you for your careful reading of our manuscript.

Algorithm 1: As it stands now this is just a sloppy code for an EnKF. The important steps that set this method apart from an EnKF are missing. Please provide a pseudo code for the whole algorithm. Also note that the output is an ensemble, not just the ensemble mean. Finally, A,Q, R and D need to be defined too.

We thank the reviewer for pointing out ways we could improve clarity of the pseudo code given by Algorithm 1. We have revised the Algorithm description so that it more concisely reflects the steps in our proposed PEnKF. We have added another step where we show that we need to estimate the modified Kalman gain matrix by solving Eq. 2. We have included the sample covariance computation S and the inverse of the result of Eq. 2, showing how they are incorporated into the PEnKF state update. We have also added definitions of A, Q, R and D to the inputs and added the ensemble output.

Algorithm 2: I suggest removing this algorithm as an EnKF is never coded up this way for high-dimensional systems. Instead, the solution is found in ensemble space.

As suggested by the reviewer, we have removed this algorithm from the paper.

Reviewer #3: All my comments have been addressed in an appropriate way. I also read over to the comments from other reviewers and I think they have addressed too.

We are gratified by the reviewer's positive comments acknowledging the responsiveness of our revisions of the paper.
